# Sub-Saharan African communities’ experiences and engagement with COVID-19 and the related control strategies in Antwerp, Belgium

**DOI:** 10.1186/s12939-023-01867-w

**Published:** 2023-03-28

**Authors:** Charles Ddungu, Lazare Manirankunda, Marie Meudec, Ella Van Landeghem, Jef Vanhamel, Deogratias Katsuva, Christiana Nöstlinger

**Affiliations:** 1 Department of Public Health, Institute of Tropical Medicine Antwerp, Antwerp, Belgium; 2 Outbreak Research Team, Department of Public Health, Institute of Tropical Medicine, Antwerp, Belgium Institute of Tropical Medicine Antwerp, Antwerpen, Belgium

**Keywords:** COVID-19, Racial/ethnic minorities, Sub-Saharan African communities, Social and economic vulnerabilities, Community engagement

## Abstract

**Background:**

Pre-existing racial/ethnic disparities in health, sustained by intersecting socio-economic and structural inequities, have widened due to the COVID-19 pandemic. Yet, little attention has been paid to the lived experiences of people in ethnic/racialised minority communities, and to the causes and effects underlying the COVID-19-related burden. This hinders tailored responses. This study explores Sub-Saharan African (SSA) communities’ needs, perceptions, and experiences of the COVID-19 pandemic and its control measures in Antwerp (Belgium) in 2020.

**Methods:**

This qualitative study using an interpretative ethnographical approach adopted an iterative and participatory methodology: a community advisory board advised on all stages of the research process. Interviews and a group discussion were conducted online, through telephone, and face-to-face. We analysed the data inductively using a thematic analytical approach.

**Results:**

Our respondents, who mostly used social media for information, struggled with misinformation about the new virus and prevention measures. They reported to be vulnerable to misinformation about the origin of the pandemic, risk of infection with SARS-CoV-2, and the prevention measures. Not only did the epidemic affect SSA communities, but to a larger extent, the control strategies did—especially the lockdown. Respondents perceived the interaction of social factors (i.e. being migrants, being undocumented, having experienced racism and discrimination) and economic factors (i.e. working in temporary and precarious jobs, not being able to apply for unemployment benefit, crowded housing conditions) as increasing the burden of COVID-19 control measures. In turn, these experiences influenced people’s perceptions and attitudes, and may have partially impaired them to follow some public health COVID-19 prevention guidelines. Despite these challenges, communities developed bottom-up initiatives to react quickly to the epidemic, including translation of prevention messages, food distribution, and online spiritual support.

**Conclusion:**

Pre-existing disparities influenced the perceptions of and attitudes towards COVID-19 and its control strategies among SSA communities. To better design support and control strategies targeted to specific groups, we need to not only involve communities and address their specific needs and concerns, but also build on their strengths and resilience. This will remain important in the context of widening disparities and future epidemics.

## Introduction

The COVID-19 pandemic has affected people around the world unequally. There is growing evidence that pre-existing racial/ethnic disparities in health, sustained by complex socio-economic determinants and structural inequities, seem to have been widened by the COVID-19 pandemic [1]. Studies in the United States (US) [2], the United Kingdom [3], Brazil [4], and South Africa [5] showed that ethnic minority groups were most affected by negative psychosocial and economic consequences of COVID-19 control measures [6].

To understand the linkages between social, racial, and health inequalities among racialised and other marginalised groups, it is useful to adopt an intersectional lens. The term ‘intersectionality’ has been used since 1989 to describe how race, class, gender, and other individual characteristics ‘intersect’ [7], and how these multiple layers of advantages and disadvantages impact health and well-being. This conceptual framework has been employed to understand, for example, how poverty, food, and housing insecurity; non-adapted education; language barriers; and migration status limit access to basic amenities including health and social care, thereby increasing health risks and directly affecting health status [8, 9]. Similarly, intersectionality highlights how different forms of stigma and discrimination may further reduce access to health and social services.

Despite this evidence about increased health disparities, there is limited knowledge about communities’ lived experiences and attributions about these problems, and to the web of causes and effects underlying COVID-19-related suffering. This hinders true engagement with communities and co-creation of strategies to address health disparities and emerging problems.

Belgium was heavily impacted by the first wave of the COVID-19 pandemic, with 30,014 COVID-19-related deaths between March and May 2020 [10]. Within Belgium, the Antwerp metropolitan region was particularly hard hit, especially at the start of the epidemic [11]. National measures focused on protecting people’s lives (i.e. in elderly care homes) and keeping hospitals afloat, while implementing lockdowns and quarantines. The public health measures were centrally steered and largely focused on informing the public about the epidemic status—as measured by reported cases, hospital admissions, and mortality—as well as on implementing control measures [12]. They targeted the general population, delivering information mainly through mainstream mass media. Yet, the Antwerp local government signalled a need for local differentiation of communication strategies, to address local population diversity. Authorities had noticed that certain racialised and ethnic minorities seemed more vulnerable to SARS-CoV-2 infection, presumably due to social and economic factors. Antwerp is a diverse city with a heterogeneous national, racial, and ethnic population [12], with almost 50% of inhabitants having a migrant background in 2020 [13]. People of Sub-Saharan African (SSA) descent represent 4.4% of the city’s population [14], although this might be an underestimation as official registers exclude undocumented and mobile people. The latter were estimated to be 21.1% of the SSA sub-population living in Antwerp city in 2016 [15]. The SSA communities in Antwerp are heterogeneous in terms of countries of origin, ethno-cultural background, official languages spoken in countries of origin, religious affiliations, length of residence in Belgium, and migration status [16]. They live mostly concentrated in the most deprived areas of the city, with one-third of all residents living in poverty [14]. A considerable number have limited knowledge of Dutch and French, two of the three official languages of Belgium.

We conducted this study to explore local needs, perceptions, and different experiences of the COVID-19 pandemic in Antwerp city from the perspective of SSA communities in Antwerp. The study particularly aimed at exploring the lived experiences of COVID-19 and the prevention and control strategies, and to examine coping strategies against these measures. Findings from this study may inform future responses to epidemic/pandemic outbreaks while considering input from racialised and ethnic minorities in order to understand and address the intersecting factors causing their vulnerabilities.

## Methods

### Study context and design

This qualitative study was part of a larger rapid assessment at the start of the COVID-19 outbreak in Antwerp among several racialised and ethnic minorities including SSA communities, Moroccan, Syrian, Turkish, and Orthodox Jewish communities. Detailed findings from the Orthodox Jewish communities [17] and issues cutting across the diverse communities [18] have been described elsewhere. Because we were interested in communities’ lived experiences, we chose an interpretative ethnographical approach [19], a method suitable to explore peoples’ perceptions of and attitudes towards COVID-19 and its control measures, and how they coped with these measures [20]. We used iterative and participatory methodologies: we conducted semi-structured interviews to explore personal experiences, and captured community perceptions and attitudes in a group discussion. We set up a community advisory board (CAB) to advise on recruitment of participants, data collection, and interpretation of findings. The input of the CAB contributed to shaping the focus of data collection and interpretation of findings.

### Sampling and data collection

Two rounds of data collection took place in SSA communities: the first from April to June 2020, during the first COVID-19 lockdown period and the second from August to September 2020, during a local resurgence of SARS-CoV-2 infections in Antwerp city. We employed convenience sampling to recruit study participants from networks of SSA socio-cultural and faith-based organisations involved in the HIV prevention field. Through these networks, we selected key persons, influential community members, and volunteers with the HIV/STI-prevention network—HIV-SAM.^1^ These study participants were selected not only because we could easily reach them during the local lockdown, but also because they had influential roles as leaders of socio-cultural and religious organisations. Through these roles, these informants had good knowledge of community structures and could identify other study participants. As influential persons in their communities, they assisted in recruiting further participants when necessary [21]. To be included in the study, participants had to identify as belonging to SSA communities in Antwerp, be 18 years or older, and be able to provide informed consent. We continued sampling using an iterative approach until code, thematic, and meaning saturation were achieved [22].

Data were collected by CD, LM, and DK, who are experienced in working with SSA communities in the HIV prevention field. We used adapted topic guides for the semi-structured interviews and the group discussion (GD), focusing on accessing information, risk perception, beliefs about and perceptions of COVID-19 pandemic, knowledge of prevention measures and coping with them, and experiences of the effects of the lockdown and COVID-19 prevention measures. We conducted the first 19 interviews and the GD digitally using the researchers’ professional and protected Zoom account, or through telephone. We conducted two additional face-to-face interviews when restrictions were gradually relaxed while respecting COVID-19 prevention measures. We collected all data in English and/or French. We audio-recorded 16 out of the 21 interviews and combined these recordings with the researchers’ notes into extensive summaries. Five interviews and the GD were not recorded due to privacy concerns or technical problems. For these, we took extensive notes and made summaries.

### Data analysis

We analysed the 16 transcribed interviews, notes of unrecorded interviews, and summaries inductively using a thematic analytical approach [23]. After familiarising with the data, the first author generated codes and themes based on the question in the topic guide, and then established an analytical framework based on the transcripts and summaries. The second and third authors reviewed the generated themes and used them for coding subsequent transcripts, thus validating the framework and resulting in preliminary themes. We shared the preliminary results with the CAB representatives from SSA communities for feedback and interpretation. They advised on the selected themes and on the focus of the interpretation resulting into final themes and narratives.

### Ethical approval

The study received ethical approval (IRB ref. 1391/20) of the Institutional Review Board (IRB) of the Institute of Tropical Medicine (ITM), Antwerp, Belgium.

## Results

Study participants came from the Democratic Republic of the Congo (*n* = 13), Burundi (*n* = 2), Cameroon (*n* = 2), Eritrea (*n* = 1), Ghana (*n* = 2), Guinea-Conakry (*n* = 1), Nigeria (*n* = 2), South Africa (*n* = 1), Somalia (*n* = 1), and Uganda (*n* = 2). The majority were male, highly educated, and Christian. Other socio-demographic characteristics of participants are outlined in Table 1.

**Table 1:** Socio-demographic characteristics of participants

Characteristics	Interview participants	Group Discussion participants
	Total (*n* = 21)	Total (*n* = 6)
**Gender**		
Men	13	4
Women	8	2
**Median age in years (min, max)**	45 (27, 71)	49 (37, 59)
**Median years of residence in Belgium (min, max)**	16 (3, 35)	17 (10, 29)
**Religion**		
Christianity	17	6
Islam	3	
Judaism	1	
**Education level**		
Secondary school	5	
Bachelor’s degree	2	
High school/university	13	6
**Employment**		
Yes	11	6
No	10	
**Position in the community**		
Leaders of community organisations	12	
Religious leaders	4	
Volunteers with the HIV-SAM	9	
Influential informal positions	2	

We identified six main themes grounded in the data, and grouped them as follows: (1) sources of information on COVID-19 and perception of its origin; (2) knowledge of and adherence to COVID-19 prevention measures and risk perception; (3) attitudes towards COVID-19 testing, COVID-19 patients, and future vaccination; (4) collateral negative effect of lockdown measures; (5) community engagement during the COVID-19 crisis; and (6) the intersecting migration-related, social, and economic disadvantages and their impact on people’s attitudes towards COVID-19 prevention measures. As a last and separate point, we report on participants’ recommendations on context-specific and culturally-relevant responses to COVID-19.

### Sources of information on COVID-19 and perception about the origin of the pandemic

Participants explained that members of their communities used various media channels depending on language, affordability, and trust in these sources. Many people from the SSA communities used social media, a few watched mainstream television, while others relied on word-of-mouth.

Social media was widely used as a source of information and space for social networking (e.g., WhatsApp, Facebook, and YouTube). WhatsApp was the most popular platform because of its affordability and user-friendliness, allowing for quick dissemination of information. People believed in the provided information mainly when they trusted the sender and network:

*“They trust most in the information that they get from us [religious leaders] when they get it on Facebook or WhatsApp groups… it’s the most trustful information for them.”* (Male religious leader, 45 years)

Participants also admitted that social media facilitated the spread of ‘fake news’ and conspiracy theories. They feared that misinformation could mislead people and discourage them from adhering to official public health guidelines on COVID-19 prevention, as two respondents explained:

*“They believe that Corona is created in a laboratory and by politicians and therefore better to be informed by WhatsApp, the internet, and other media channels that oppose the system […].”* (Male participant, 64 years)

*“This [misinformation] is the biggest challenge*, *lots of false information are shared online, for example, that African people will be used for experiment purposes. This makes people afraid even to go to see doctors when they feel sick.”* (Male community leader, 42 years)

Furthermore, participants felt that misinformation aggravated the fear and anxiety that people were already experiencing. Frequently-mentioned conspiracy theories were blaming, among others, China, Bill Gates, pharmaceutical companies, Western politicians, and governments to have caused the outbreak. The following examples highlight some opinions on conspiracy theories that many people believed in:

*“I mean, there are even racist conspiracy theories or thoughts, that people think that it [COVID-19] was created, you know, to kill off some races and no other races, there’s even ageism where they are saying that it was designed to kill off the old and the weak and as a biological weapon.”* (Female participant, 30 years)

*“They say that it (COVID-19) is a disease that does not attack Black skin but when you look at what is happening in France, there is the number of Congolese who have succumbed to the pandemic. They say that it is the white people who kill them to take their organs and sell them. This information is spread by social media platforms and [some] African television and everyone tends to believe it.”* (Female leader of a social-cultural organisation, 63 years)

*“… There’s also [a belief] that it’s created by China to take over the world*.*”* (Female participant person, 39 years)

*“Most of the people you meet will tell you that it is God who is angry, we have done a lot of stupid things and God is punishing the world.”* (Male leader of a socio-cultural organisation, 44 years)

Other participants expressed their struggle with information overload emerging from social media and the difficulty they faced to handle it:

*“In my opinion, people are lost, I am lost too. We hang on to social media to get information but we do not master it [the information] because some are contradictory […].”* (Female leader of a socio-cultural organisation, 63 years)

Few people who got information from television reportedly watched BBC, CNN America, and the Francophone Belgian and French television channels. Others got information from online television channels from their countries of origin. The Flemish-language television channels were less watched. The following quote illustrates how language and trust determined the choice of TV channel:

*“Yah, because they don’t speak the language [Flemish], it is very hard for them. So, they always listen to the BBC Somalian news […] because they know it is based in London, they are very satisfied of getting accurate news.”* (Female teacher of a social integration course, 31 years)

### Knowledge of and adherence to COVID-19 prevention measures and risk perception

In general, participants rated knowledge about COVID-19 prevention measures among their fellow community members as high. Many people were believed to be aware of the severity of COVID-19, and reported that people in their communities tried their best to adhere to the prevention measures:

*“I think that during this pandemic, everybody knows what they must do. […] We thought we have to take care of our lives. […]. We have informed each other, and I think that everyone understood the importance of this.”* (Male leader of a socio-cultural organisation, 44 years)

*“In general people are informed that there is a virus that is killing people and how we can avoid getting it…, [for example] we are no longer going for night prayers, although missing this causes lot of isolation and depression.”* (Female participant, 53 years)

However, cases of limited knowledge and trivialisation of the epidemic were also reported:

*“There are those who are well-informed but who trivialise the information and those who are ill-informed because of their environment, to which religious beliefs are part…”* (Female leader of a socio-cultural organisation, 63 years)

Although most people reportedly understood the importance of the imposed prevention measures, some encountered barriers to adhere to them. Two specific challenges were highlighted, namely crowded homes and difficulties to refrain from social contact:

*“These [prevention measures] are difficult for us. This is mainly due to small spaces we have. We live on top of each other. Those with two or three children have it very difficult.”* (Female participant, 53 years)

*“Yeah, according to the culture and tradition, this is the most difficult moment for [X]*^2^*people, because it’s difficult to keep away from other people, they are very social, […] and shake hands and hug a lot.”* (Community leader, 38 years)

*“*W*hen they come to [X social integration centre],*^3^* they must wear a face mask, but they [still] come to hug me because they haven’t seen me for so long. During the lockdown, they only heard my voice. So, they came sometimes from behind and hugged me […] ‘Ah, [they said] we don’t care about corona. We must give you a hug, [because] we missed you’.”* (Female teacher of a social integration course, 31 years)

Regarding risk perception, people had opposing views on the potential risk of acquiring COVID-19. On the one hand, some thought that they were not at risk of contracting COVID-19 because of a predisposition linked to their African origin:

*“We Africans are stronger, for example, we support malaria and other diseases*.*”* (Female assistant church leader, 31 years)

This idea, which was reinforced by stories on social media, was strongest at the early phase of the pandemic but faded as the pandemic evolved:

*“At the beginning, there was a wrong belief that Africans cannot be affected by the virus.”* (Male participant, 58 years)

*“People used to say ‘this is a European disease; it doesn't concern us’. However, the more the pandemic evolved, the more they started seeing that it affected Africans as well, but at the beginning, they didn't take it seriously.”* (Male leader of a socio-cultural organisation, 44 years)

On the other hand, some people considered themselves equally at risk like any other person in Belgium:

*“I know, it [COVID-19] is not only for Black people, but for White people as well, but there are still people with this mentality that ‘I can’t get it’.”* (Male leader of a social-cultural organisation, 47 years)

### Attitudes towards COVID-19 testing, COVID-19 patients, and future vaccination

#### Testing for COVID-19

In general, respondents reported that people accepted COVID-19 testing. However, during the first wave, testing was accessible only to people with symptoms. Participants questioned this limitation:

*“The most effective remedy for this virus is testing. If we test everyone, then you will know. There will be transparency, you will know the state of your body.”* (Male participant, 64 years)

Targeted testing was not seen as a favourable option because participants considered it discriminatory and potentially stigmatising:

*“I think that [X]*^4^*people will hide [from testing] if I consider the current debate on vaccines. They think that the idea behind screening is to test the vaccine on Africans everywhere as guinea pigs. They won’t accept it [the COVID-19 test].”* (Leader of a socio-cultural organisation, 63 years)

Despite this relatively positive attitude towards generalised COVID-19 testing, hesitancy was also noted due to misinformation on social media. Rumours were circulating, for instance, that some COVID-19 tests were contaminated. Other people suspected a hidden agenda behind testing and fear of targeted testing was alluded to as a potential factor of stress and anxiety during that period:

*“People are afraid of screening because they ask themselves: ‘what's next?’ […] We think there is a Machiavellian plan behind it, many people will refuse it. It is believed that the virus was man-made and there are doubts about the current approach. Screening motives are not clear, and the question is: ‘after the screening, what will happen?’.”* (Male religious leader, 44 years)

Opinions on contact tracing were diverse and sometimes contradictory with those expressed on COVID-19 testing and its acceptability as a future mass test. Some participants thought that contact tracing would be beneficial in stopping the spread of COVID-19, as one respondent noted:

*“Personally, I think it [contact tracing] is good because you have to protect others [...]. I think they [people] understand it because the disease is very contagious. You have to protect the society.”* (Community leader, 66 years)

Others did not trust contact tracing because they feared it would intrude their privacy and because they also suspected a ‘hidden agenda’:

*“I don’t think there’s a need for large scale testing and case finding (contact tracing) […]. I think that ship has sailed, and if we start doing it now, what are the reasons behind it […].”* (Female participant, 39 years)

#### COVID-19 patients

A recurring narrative in the interviews was the potential stigmatisation of COVID-19 patients. Participants reported different experiences on this matter. On the one hand, people cured of COVID-19 were accepted and supported, on the other hand some also reported cases of stigmatised patients due to fear and uncertainty about this new disease:

*“Normally, when you have been ill [of COVID-19], everyone must avoid you, even if you are cured. But there are testimonies that people [those who have been cured] make and these give a bit of hope and are encouraging.”* (Male leader of a socio-cultural organisation, 44 years)

*“When this thing [COVID-19 pandemic] started, there was one of our members, an elderly man, about 73, who called me. He was coughing and went just for a routine check-up, because he is diabetic. They had to quarantine him at the hospital. Somebody heard that he was in the hospital and was tested for COVID-19, and people were scared to meet him.”* (Male religious leader, 49-years old).

#### COVID-19 vaccines

COVID-19 vaccines were not yet available at the time of data collection, but we wanted to anticipate their future acceptability among SSA communities. Respondents reported three opinions: outright rejection, hesitancy, and relative acceptability. Anticipated rejection of the vaccine was particularly fuelled by the controversial proposal of two French physicians, who had suggested that a COVID-19 vaccine should first be tested in Africa because of the perceived high exposure to COVID-19 [24]. Participants said that this televised debate had led members of their communities to feel racially profiled and be considered as guinea pigs. This controversy reportedly provoked a lively conversation on social media, in which some people set out to convince others to refuse any future COVID-19 vaccine:.

*“It will be a complicated situation given thousands of negative statements about the vaccine on black skin. I myself, speaking to you, will not accept. There are some who are talking to create buzz in our social networks but there are also trusted people who are warning Africans not to accept any COVID-19 vaccine. Why should we accept?”* (Female leader of a socio-cultural organisation, 63 years)

*“People won't accept it [the vaccine] because they think they are going to be injected with a chip that will expose them to be watched wherever they are […]. But they think it will be imposed on us, for example, at the airports.”* (Male religious leader, 45 years)

Participants who anticipated vaccine hesitancy among community members, but who were in favour of the vaccine, argued that people needed to be convinced. They highlighted that community leaders should be involved in the process because people trusted them:

*“The vaccine can be accepted but with hesitation. It can, however, be accepted if leaders do it first in public. Community leaders will have an important role to play because people trust them. So, community leaders should be convinced to take the vaccination first.”* (Male community leader, 42 years)

### Lockdown measures’ perceived collateral negative effects

Participants agreed that people in their communities had suffered a heavy toll from the collateral damage of COVID-19. They felt that COVID-19 negatively impacted people’s lives in different ways, including economic and psychosocial consequences, both individually and collectively. Participants considered the economic impact as the most severe. Several respondents reported that many people in their communities worked in the informal sector or on short-term contracts and were therefore unable to apply for formal financial support installed by the government. Moreover, since short-term contracts were not possible during the lockdown and opportunities for informal work had decreased, survival for these people became more difficult:

*“For migrants, especially those without papers, corona is a catastrophe, a threat to survival. There is a big effect on income, even for employed people.”* (Male leader of a socio-cultural organisation, 45 years)

*“My experience is positive […]. But I have papers, those who don’t have it [paper] is another story because they cannot go anywhere [out]. Lucky enough nobody has been infected [by COVID-19]. I have not heard any plan to help people without papers. If another outbreak comes, they should say that anybody regardless of their residence status can be helped.”* (Female participant, 53 years)

The subsequent inability to send remittances to families in their countries of origin reportedly caused anxiety for some:

*“Africans have always managed to survive and help their relatives back home by sending money, but it was no longer possible because even Western Union was closed*.*”* (Female participant, 53 years)

In addition, the closure of social gathering places, including churches, had a significant negative impact on people’s social and spiritual lives:

*“Two months ago, according to the Orthodox Church, for example, it was Easter, but they could not celebrate it and that was difficult.”* (Community leader, 45 years)

*“For me, Ramadan was very hard, because half of it, I was alone at home with my family. Ramadan shouldn’t be like that. Ramadan means coming together, eating together, celebrating together […] and I didn’t feel any togetherness.”* (Female participant, 27 years)

### Community engagement during the COVID-19 crisis

Participants reported various bottom-up initiatives in response to the COVID-19 crisis, particularly in well-organised communities. For example, support from organisations and church leaders came mainly in the form of translation and dissemination of prevention messages and official government communications into other languages used in different communities (English, French, or other native languages), administrative support (assistance with online registration in order to receive financial support from the federal government), engaging with false information about the pandemic, psychosocial support (calling isolated persons, counselling, etc.), and food distribution:

*“We have so many WhatsApp groups. So, by any change of [official] information we send it to the different groups. For information in Dutch, I must convert it into English or in another native language, I send to the group in their language. If the pastor finds information like extension of the lockdown or a letter from the government on corona, he forwards it.”* (Female assistant church leader, 30 years)

Some participants indicated that the crisis had led to community and religious leaders taking on work that is normally done by social workers, as one respondent testified:

*“*A*t the beginning it was also difficult for them to ask for their unemployment benefit. Some of them were late to do that [...] and we organised a group, and helped them online, to fill-in a form and to send it online to the trade union.”* (Male community leader, 41-years-old)

Other forms of support were psychosocial, such as reaching out to the lonely and elderly to provide moral support, a feeling of community, and encouraging all members to keep in touch with each other, as the following example shows:

*“I take initiatives and call them. I know that people are living in such [lonely] situations, so I call them to make sure that they are also still safe, and that they are following the rules too […].”* (Male religious leader, 45 years)

### Intersecting migration-related, social, and economic disadvantages and their impact on people’s attitudes towards and adherence to COVID-19 prevention measures

Looking at the results with an intersectional lens, we notice the combination of multiple migration-related, social, and economic disadvantages, which were perceived as significant in shaping people’s experience of the pandemic and its control measures. In turn, these experiences influenced people’s perceptions and attitudes and partially impaired them from following some public health COVID-19 prevention guidelines.

**Fig. 1:**
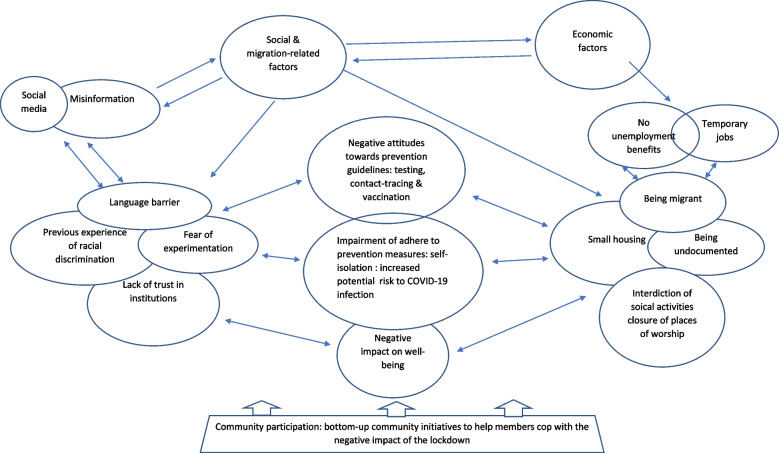
Overlapping layers of social and economic disadvantages and their impact on people’s perceptions of and attitudes towards the COVID-pandemic and its control measures

Social issues relevant for many in our study population were: being migrants, living in small houses, being undocumented, perceived racism, and discrimination. The lack of trust in mainstream media channels and public health institutions, which might have been exacerbated by the above factors, and a common language barrier, led many to rely mainly on social media, which exposed them to a lot of misinformation. An example of such interlinkage is the previous experience of stigma and discrimination and distrust in healthcare institutions in media is that a news item about a proposed clinical trial of COVID-19 vaccine in Africa was perceived as racist, and capitalised by social media. Results illustrate the extent to which misinformation on social media also contributed to the reported negative attitudes towards COVID-19 public health prevention measures. Economic disadvantages included working in temporary and precarious jobs and not being able to apply for unemployment benefit, which significantly affected people’s already fragile livelihoods. The above socio-economic and migration-related factors plus the interdiction of social gatherings and closure of places of worship where people got psychosocial and spiritual support combined to negatively impact people’s psychosocial well-being significantly. In Fig. 1, we have mapped the different pathways of how the interaction of migration-related, social, and economic determinants shaped people’s experience of the pandemic. Reportedly, participants perceived the impact of COVID-19 on their own communities as ‘more negative’ than on the general population.

### Participants’ recommendations

For context-specific and culturally-relevant responses to COVID-19, participants recommended using multiple communication channels adapted to people’s life situations. They also recommended taking into consideration people’s capabilities including their health and digital literacy. They also advised that their community leaders should be involved in developing and implementing control and prevention strategies related to COVID-19. This recommendation is in line with the guidance on community engagement for public health events caused by communicable disease threats in the European Union/ European Economic Area (EU/EEA) recommending “viewing the community as a partner and a resource for optimising preparedness planning, response and recovery” [25].

To effectively address the information and prevention needs of the population, respondents advised tackling misinformation and rumours about COVID-19. In addition to this proposal, it will be important in future research to understand the patterns of this phenomenon and the reasons why many of our participants believed in misinformation. To combat COVID-19-related misinformation, participants also suggested training people in the use of social media, enabling them to distinguish between correct and false information. Such trainings should be given taking into account their capabilities.

Regarding COVID-19 testing at the time of data collection, participants recommended expanding and simplifying COVID-19 testing protocols, making it less bureaucratic and offered to all regardless of legal residence status. Finally, recognition and support for community-based initiatives, as well as financial support for those working in the informal sector, were suggested as essential to achieving equitable prevention outcomes so that no one is left behind.

Concerning stigmatisation of COVID-19 patients, participants recommended that the variation in people’s attitudes towards COVID-19 patients and the impact on prevention behaviours needed to be monitored and explored further to ensure that no one is excluded.

## Discussion

This article presents SSA communities’ lived experiences in the Antwerp metropolitan area with the COVID-19 pandemic and the related control strategies, and the engagement of these communities in responding to different emerging challenges during the first phase of the outbreak in 2020. The main findings relate to information sources, language barriers, and COVID-19-related misinformation and mistrust related to previous experiences of racial discrimination, and intersectional factors of vulnerability partially challenging adherence to control and prevention strategies.

Study participants reported that people in their communities, who mostly used social media as a source of information, struggled with misinformation about the new virus and prevention measures. The responses illustrate that the choices of information channels were related to language and trust in institutions, including mainstream media. The flow of misinformation about COVID-19 on social media increased confusion and further affected trust in institutions.

The study results showed that many people were vulnerable to misinformation, but we did not identify specific determinants of people’s susceptibility to misinformation. To that end, future research should look into factors specifically relevant to SSA communities to guide tailored prevention. Some studies conducted in the general population to understand people’s susceptibility to misinformation and conspiracy theories relating to general topics and COVID-19 revealed several factors, such being older, having strong traditional value systems, and a self-reported minority social status [26–28].

Perceptions of the risk of infection with SARS-CoV-2 were equally diverse and changed as the epidemic evolved. Initially, people’s low-risk perception was influenced by information they received from their countries of origin where the pandemic had not yet hit hard. This information, via social media, led some to believe that they were not at risk, simply because they were of African descent. This idea faded as the pandemic evolved. A French study found similar evolutions in risk perception over time [29]. Similarly, perspectives on preventive measures such as testing, contact tracing, and future vaccination changed. Initial reactions to mass testing were positive for some, particularly in the case of COVID-related symptoms. However, participants strongly rejected targeted testing or a ‘key populations approach’ [30], which they perceived as racial stereotyping and stigmatising. Previous experiences of cross-sectional stigma and discrimination, as documented in a study among Belgians of African descent [31], may have played a role in this rejection. Participants generally perceived COVID-19 patients as facing the risk of being stigmatised, although few saw social exclusion as a real danger. Quite a few participants anticipated that fear and uncertainty about this new disease and lack of sufficient knowledge and misinformation would increase the risk of stigmatisation. Studies on HIV, Ebola, or Zika epidemics also referred to stigmatisation of ethnic and racialised minorities [32, 33], confirming the importance of adapting stigma-informed approaches in outbreak responses. Specific to COVID-19 and stigmatisation of patients, studies from other countries also acknowledged the reality of stigma related to COVID-19 [34, 35]. The acceptability of a future COVID-19 vaccine was a controversial issue. The incident of French researchers suggesting that new vaccines should be ‘tested’ in Africa illustrates the widespread and long-term impact of single events on trust and attitude, especially if such messages are capitalised upon by interested groups on social media who link them to their experiences of racism in other forms. The mistrust of institutions by a racialised minority group based on earlier experiences of racism seen in our study is comparable to the findings in other studies [36]. A US-based study, for example, highlighted that prior experiences of racism, discrimination, and ‘fear of experimentation’ contributed to mistrust in the healthcare systems by some African Americans, which in turn negatively impacted their acceptance of and willingness to seek healthcare [37].

Our study also showed the negative collateral economic and psychosocial impact of the COVID-19 lockdown during the first wave. For example, housing conditions and social norms made it difficult for people to follow social distance, quarantine, and isolation guidelines. This is in line with findings from an Organization for Economic Co-operation and Development (OECD) study highlighting that some vulnerable groups, such as people with a migration background, are at higher risk of both COVID-19 and its negative effects “due to a range of vulnerabilities such as higher incidence of poverty, overcrowded housing conditions, and high concentration in jobs where physical distancing is difficult” [38]. Our results illustrate the findings from different countries showing that COVID-19 exacerbated health disparities between migrant and ethnic and racialised minorities and the general population, as study participants reported that previously-existing structural vulnerabilities were enhanced [1, 2, 5]. In a similar manner, SSA communities in Antwerp were not only affected by the epidemic, but also—and to a larger extent—by the control strategies, especially the lockdown. People without residence permits were among the most affected ones, economically and socially, but also psychologically and spiritually. Due to lockdown-related increased police controls, they isolated even more than migrants with residence status due to the fear of expulsion. However, all study participants suffered severely from the closure of social and spiritual venues, because such venues have a crucial function in addressing people’s concerns, questions, and needs, especially in times of crisis. The severe negative impact of COVID-19 and the related control measures, such as lockdowns, on ethnic/racialised minorities including immigrants has been documented by a study in France [6]. In another study, the already fragile life conditions of undocumented migrants were reported to have been worsened by the COVID-19 pandemic control measures [39].

Despite the COVID-19-related challenges we have examined, the pandemic also provided important lessons about social resilience and coping strategies of SSA communities in Antwerp. Our study findings highlight that these communities demonstrate a considerable degree of community engagement. For example, community and religious leaders improvised ad hoc solutions to challenges, from filling-in knowledge and information gaps among their members to developing creative solutions to mitigate the multi-layered impact of the first lockdown measures. Previous outbreak studies have highlighted the importance of community engagement in outbreak response to achieving sustainable and inclusive prevention outcomes [40, 41]. The European Centre for Disease Prevention and Control also recommended community engagement during all phases of epidemic response [42]. Despite this evidence, participants in our study saw no recognition of their communities as partners in the COVID-19 response during the first lockdown.

### Limitations

This study has several limitations. First, data stem mostly from the first lockdown period, thus the findings do not cover subsequent evolutions. Second, most of the interviews were conducted with key informants who reported on behalf of their communities. Due to the lockdown restrictions, it was difficult to enter communities and gather lived experiences of people who did not have leadership function in their respective communities. This led to a narrow selection of study participants, limiting the findings’ generalizability to socially and economically more-vulnerable participants who may have told different stories. This may have biased our results. Third, most data (interviews, GD, and feedback from the CAB) were collected online. Consequently, only digitally-literate people with access to the internet participated. We thus missed out on digitally-illiterate people with relevant information on structural vulnerabilities. Some participants experienced problems due to poor internet connection or were stressed because it was their first online interview. In addition, we missed body language and spontaneity of participants, limiting the depth of the conversation. Finally, some participants doubted the confidentiality of an online conversation, which may have caused them to be reserved regarding sensitive issues. These issues may have hampered the quality and richness of the data collected but were mitigated as much as possible through professional interviewing skills and through past work experience of researchers with participants.

## Conclusion

Study participants perceived SSA communities in Antwerp as significantly negatively-affected by the COVID-19 pandemic, especially by the related control measures. Pre-existing and intersecting social, economic, and migration-related disadvantages were found to be important factors negatively affecting communities’ perceptions of the pandemic, their attitudes towards and adherence to prevention strategies. For outbreak control strategies to be acceptable, feasible and thus effective, public health professionals and policy makers need to understand these intersections and their effects especially on groups in society living in precarious circumstances. This means outbreak responses have to address the structural drivers of health inequalities to effectively reach racialised and ethnic minorities. With the prolonged continuation of the COVID-19 epidemic and widening disparities, it is urgent to involve racialised and ethnic minority communities in response strategies that not only address their needs but also build on their strengths and agency.

## Data Availability

All relevant data supporting the findings of from this study are included in this published article. The complete datasets generated and analysed during the study i.e. interview transcripts and group discussion/notes/summaries are not publicly available because they contain information that can be classified as confidential. Pseudonymised data are available from the corresponding author and can be given on request for substantial reasons.

## Footnotes

1 The HIV-SAM project promotes sexual health and prevention of HIV/STIs among African communities in Flanders, Belgium. The project uses a participatory approach by working together with community and religious leaders, intermediary organisations, and individual volunteers to accomplish this task. For more information, see: http://www.hivsam.be/

2 Ethnicity removed to ensure anonymity of participant.

3 X is one of the centres for social and civic integration for newcomers in the city of Antwerp. We have used “X” to ensure the participant’s anonymity.

4 Country of origin removed to ensure anonymity and confidentiality.
